# Frozen agarose–assisted tissue preparation for stimulated Raman histology enables rapid intraoperative assessment of peripheral nerve integrity

**DOI:** 10.1016/j.bas.2026.106174

**Published:** 2026-07-16

**Authors:** Felix Schoeffing, Marc Hohenhaus, Jürgen Beck, Christoph Scholz, Jakob Straehle

**Affiliations:** a Department of Neurosurgery, University Medical Center Freiburg, Germany; b Center for Advanced Surgical Tissue Analysis, University Medical Center Freiburg, Germany

**Keywords:** Stimulated Raman histology, Traumatic nerve injury, Peripheral nerve repair, Neurosurgery, Intraoperative imaging

## Abstract

**Background:**

Intraoperative assessment of peripheral nerve viability during traumatic nerve repair is predominantly based on the surgeon’s subjective macroscopic criteria. Frozen section histology can provide objective information, its routine, repeated use is however limited by processing time. Intraoperative Stimulated Raman Histology (SRH) enables rapid, label-free imaging of native tissue. Current intraoperative SRH workflows rely on squash preparations that do not adequately preserve peripheral nerve microarchitecture.

**Objective:**

To establish a rapid tissue preparation protocol that preserves true cross-sectional nerve architecture and investigate the feasibility of SRH for the assessment of peripheral nerve resection margins.

**Methods:**

Peripheral nerve tissue from 15 patients was analyzed using SRH. Various tissue preparation strategies were systematically assessed, including freehand and guided sectioning of native, fixed and agarose embedded short-term frozen peripheral nerve samples. Workflow duration and image quality were evaluated descriptively.

**Results:**

Freehand sectioning resulted in substantial architectural distortion, preventing reliable evaluation of fascicular organization. Guided sectioning improved thickness control but remained limited by tissue deformation during the cutting process. Agarose embedding followed by 1-min dry ice exposure enabled reproducible generation of 500 μm cross-sections with well-preserved fascicular architecture and minimal artifacts in SRH. Tissue preparation required 3.5 min.

**Conclusion:**

Frozen-agarose assisted tissue preparation for SRH (FA-SRH) is feasible and compatible with surgical workflows, providing rapid visualization of peripheral nerve microarchitecture. Therefore, FA-SRH represents a promising complementary tool to support objective intraoperative decision-making during peripheral nerve repair surgery.

## Highlights

• FA-SRH enables rapid visualization of peripheral nerve microarchitecture within 6 to 7 min without fixation or cryosectioning.
• FA-SRH enables objective intraoperative assessment of nerve stump viability beyond subjective macroscopic criteria.
• Rapid FA-SRH-based visualization may support objective intraoperative decision-making during sequential nerve stump resection and repair.

## 1 Introduction

Traumatic peripheral nerve injuries pose a significant clinical challenge with substantial functional and socioeconomic impact, predominantly affecting patients of working age (Aman et al., 2022; Dunn et al., 2021; Kim et al., 2004a, 2004b). Functional outcome critically depends on the severity of axonal damage and preservation of the connective tissue framework. According to the Seddon and Sunderland classifications, surgical intervention is primarily required in higher-grade lesions involving disruption of axons and endoneurial or perineurial structures (Seddon, 1942; Sunderland, 1951).

Peripheral nerve repair frequently requires resection of neuromatous or non-viable segments prior to reconstruction. In current clinical practice, nerve stump viability is assessed by sequential trimming based on visual and tactile criteria, rendering the extent of resection largely subjective and dependent on surgical experience (Moore et al., 2015). Rapid, objective intraoperative methods for microstructural nerve assessment are therefore needed. Frozen section histology can support stump evaluation. However, its routine use is limited by preparation time, workflow disruption, and poor suitability for repeated sampling (Kim et al., 2015; Novis et al., 1997), despite evidence that fascicular disorganization and nerve sheath alterations distinguish injured from healthy nerve tissue (Murji et al., 2008).

SRH is a label-free optical imaging technique that enables near real-time, histology-like visualization of native tissue without fixation or staining by exploiting vibrational contrast of lipid- and protein-rich structures (Orringer et al., 2017). In neurosurgical oncology, SRH has shown diagnostic performance comparable to frozen section analysis with markedly reduced turnaround times and has been successfully combined with automated image analysis (Hollon et al., 2020; Straehle et al., 2022). However, the current intraoperative workflow for SRH relies on thin squash preparations (225 μm), which, depending on the size of the sample, can disrupt the cytoarchitectonic structure. While suitable for neuro-oncological applications (Orringer et al., 2017), this approach may compromise the preservation of fascicular organization and connective tissue compartments, which are critical for the evaluation of peripheral nerve microanatomy. Consequently, direct transfer of established SRH workflows (Neidert et al., 2022) to peripheral nerve surgery is not feasible without adaptation of tissue preparation.

The first study applying SRH to peripheral nerve tissue demonstrated that SRH can visualize myelinated axons and allow for the quantitative assessment of myelin content (Wilson et al., 2022). This approach relied on conventional cryosectioning to generate ultra-thin tissue slices (∼70 μm) and a modification to the microscope slides. Cryosectioning is time consuming and requires a specialized cryostat which is suboptimal for an intraoperative application.

To this end, we aimed to develop a simple, rapid, and reproducible method of tissue preparation that leverages the speed of SRH while minimizing preparation-induced artifacts of peripheral nerve microarchitecture, with the goal of enabling intraoperative evaluation of peripheral nerve resection margins during traumatic nerve repair surgery. For the development and refinement of this method, peripheral nerve tissue was obtained from a variety of surgical procedures including non-traumatic cases. This approach allowed iterative refinement of the workflow in a setting closely reflecting routine practice.

## 2 Materials and methods

### 2.1 SRH imaging platform

SRH imaging was performed using the NIO Laser Imaging System (Invenio Imaging, Santa Clara, CA, USA), installed within the operating room complex and operated by trained laboratory technicians or neurosurgeons. The system enables automated acquisition of SRH images from native tissue samples. Image acquisition time varied according to the selected field of view, ranging from 2.0 min for a 1.7 × 1.8 mm^2^ area to 11.5 min for a 4.7 × 4.8 mm^2^ area.

### 2.2 Tissue samples

SRH feasibility and workflow optimization were evaluated using peripheral nerve tissue obtained from 15 patients. Samples comprised peripheral nerves from different sites and included traumatically and postoperative altered nerve tissue as well as macroscopically intact nerve segments from diagnostic nerve biopsies. Pathological conditions varied between patients, reflecting the heterogeneity encountered in clinical peripheral nerve surgery (Table 1). Consequently, some of the included specimens originate from clinical scenarios in which intraoperative histological assessment is not routinely performed or does not directly influence surgical decision-making (e.g. neurectomy in cases of treatment-resistant meralgia paresthetica (Scholz et al., 2023)). All analyzed samples consisted exclusively of nerve tissue that was resected as part of the planned surgical procedure and would otherwise have been discarded. No additional tissue was removed for study purposes. The use of this material was approved by the local ethics committee and conducted in accordance with informed patient consent.

**Table 1:** Sample overview.

Sample	Material	Pathology
*Freehand sectioning*		
1	Tumor-bearing nerve fascicle, tibial nerve	Schwannoma, CNS-WHO-I°
*Simple guided sectioning*		
2	Residual of sural nerve interposition graft	Traumatic cutting injury of median nerve
3	Sural nerve biopsy	Amyloid neuropathy
4	Lateral femoral cutaneous nerve (LFCN)	Meralgia paraesthetica
5	Saphenous nerve, infrapatellar branch	Post-surgical saphenous/infrapatellar neuralgia
6	LFCN	Meralgia paraesthetica
7	Saphenous nerve, infrapatellar branch	Post-surgical saphenous/infrapatellar neuralgia
8	Fascicle of dorsal root C8	Intradural lipoma C7/T1
9	LFCN	Meralgia paraesthetica
10	Plantar interdigital nerve	Morton neuroma Digg. 2 + 3
11	Fascicle of dorsal root C5	Meningioma CNS-WHO-I° C4/5
*Guided sectioning, formalin-fixation (72 h)*		
12	Spinal nerve T5	Bone metastasis from breast carcinoma at T5
*Guided sectioning, agarose embedding and freezing (*2 min*)*		
13	Sural nerve biopsy	Chronic neuropathy with signs of axonal damage and discrete signs of remyelination
*Guided sectioning, agarose embedding and freezing (*1 min*)*		
14	Piece of sural nerve interposition graft	Neuroma of superficial peroneal nerve
15	Resection margins of peroneal nerve stumps	Traumatic lesion of peroneal nerve

### 2.3 Tissue preparation strategy

Initial approaches included freehand sectioning of native nerve tissue without stabilization and preparation of squash specimens on the SRH microscopy slides (Nio Slide, Invenio Imaging, Santa Clara, CA, USA). Freehand sectioning of the nerve specimen was performed using a disposable scalpel blade (Feather, size 11; Feather Safety Razor Co., Ltd., Osaka, Japan).

Subsequently, guided sectioning using a Mouse Spinal Cord Matrix (Ted Pella Inc., Redding, CA, USA; #15171), designed to generate defined cross-sectional thicknesses of 500 μm, was introduced to improve section thickness control. Commercially available razor blades were used for tissue sectioning.

**Fig. 1:**
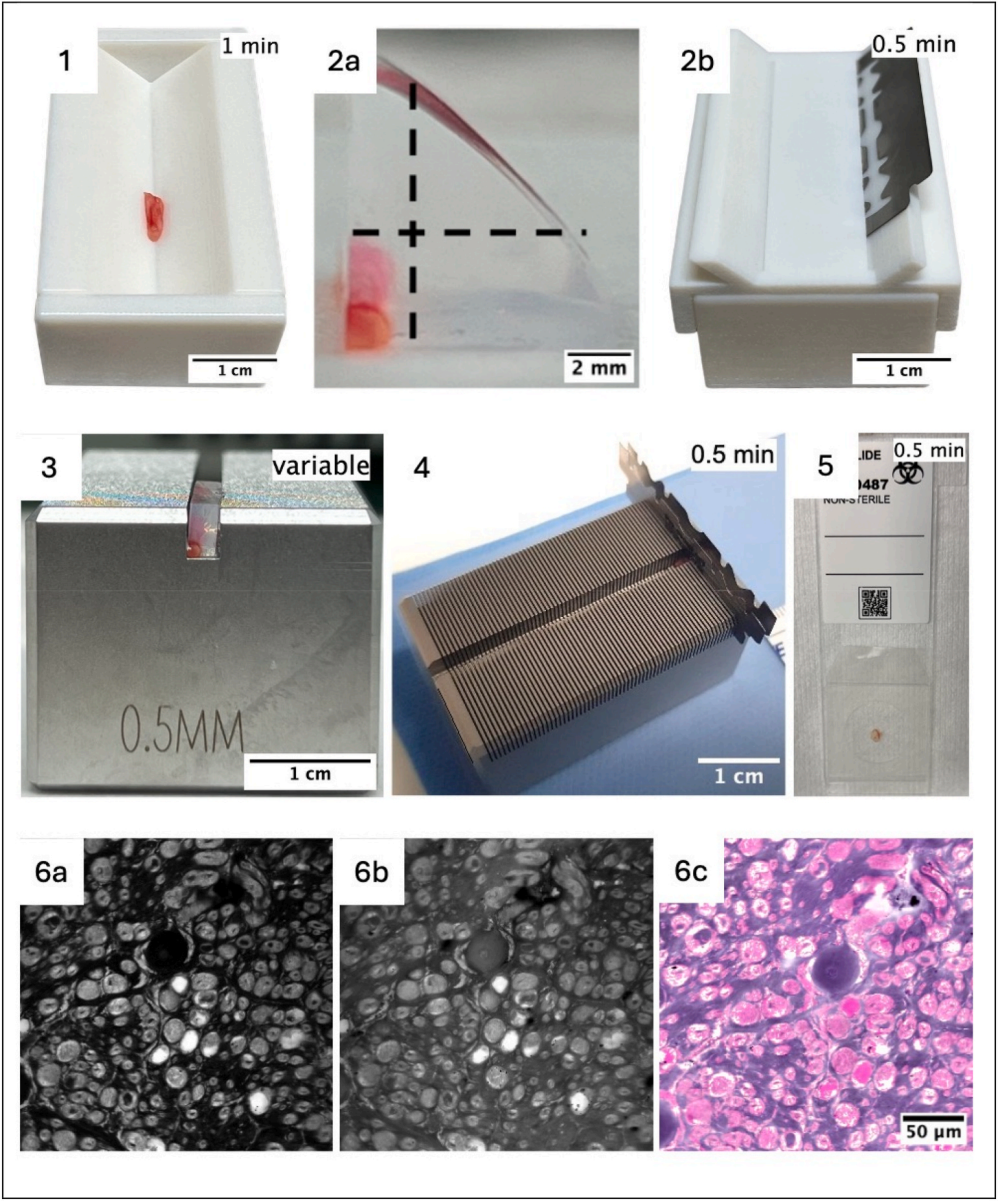
Frozen-agarose tissue preparation for SRH (FA-SRH). Step *1* agarose-embedded native nerve specimens in a triangular casting mold with a right angle. Step *2a + b* guided trimming of the nerve–agarose block. Step *3* placement of the trimmed nerve-agarose block into the cutting matrix with parallel cutting slots spaced at 500 μm and exposure to dry ice. Step *4* guided tissue sectioning with a razor blade. Step *5* placement of 500 μm-sectioned tissue slice on a NIO slide and squash preparation with the coverslip. Step *6* SRH images were acquired at two Raman shifts (2845 cm^−1^, lipid channel, *6a*; 2930 cm^−1^, protein/DNA channel, *6b*) and computationally rendered into a virtual H&E-like image for analysis and clinical interpretation *(6c)*.

To further enhance cutting precision, different tissue hardening strategies were assessed. Tissue rigidity was increased either by formalin fixation (72 h in 4% formaldehyde) or embedding in agarose and brief exposure (1 to 2 min) to dry ice (FA-SRH, Fig. 1).

For FA-SRH native nerve specimens were embedded in ultra-low gelling agarose (3% w/v; #A2576-5g, Sigma-Aldrich, St. Louis, MO, USA) using a custom-designed, 3D-printed, V-shaped casting mold with a right angle to ensure consistent specimen orientation (Fig. 1, Step 1). Agarose aliquots were heated briefly until boiling in a commercially available microwave. The molten agarose was subsequently maintained at 37 °C under gentle agitation (300 rpm) using a thermomixer (Thermomixer Comfort, ST124223, Eppendorf, Hamburg, Germany) to prevent premature solidification.

After agarose solidification at room temperature (1 min), the nerve–agarose block (Fig. 1, Step 2a) was trimmed using a custom-made, 3D-printed cutting guide to match the dimensions of the cutting matrix (Fig. 1, Step 2b).

This geometry enabled reproducible placement of the specimen into the corner of the cutting matrix (Fig. 1, Step 3). After placement of the agarose-embedded nerve specimen into the cutting matrix, the matrix was positioned on dry ice (solid carbon dioxide, stored at −80 °C) within an insulated, closable container. Exposure time was precisely controlled using a standard laboratory timer. Based on iterative optimization in three samples, the duration of dry ice exposure was systematically reduced from 2 to 1 min. The temperatures reached inside the insulated container were −66.7 ± 5.8 °C for 1 min and −82.0 ± 6.1 °C for 2 min exposure to dry ice, respectively.

Following partial freezing, the agarose-embedded nerve specimen was sectioned into serial slices with a target thickness of 500 μm using the cutting matrix (Fig. 1, Step 4).

For SRH imaging, a single 500 μm-thick tissue slice was then transferred using fine-point forceps (e.g., Fisherbrand™, Thermo Fisher Scientific, Waltham, MA, USA; #12-000-131) to an NIO slide and positioned centrally. Excess agarose surrounding the nerve tissue was removed using fine forceps. In accordance with the standardized NIO imaging workflow, a coverslip was then gently applied to flatten the tissue slice to an effective thickness of 225 μm (Fig. 1, Step 5). The slide was transferred to the motorized stage of the NIO Laser Imaging System. Raw stimulated Raman scattering images were acquired as sequential image strips at two Raman shifts: 2845 cm^−1^ corresponding to lipids (CH_2_ stretching mode; Fig. 1, Step 6a) and 2930 cm^−1^ corresponding to protein and DNA (CH_3_ stretching mode; Fig. 1, Step 6b). These raw channels were subsequently computationally combined and rendered using a virtual hematoxylin-and-eosin (H&E)-like color scheme (Invenio Imaging) to facilitate intuitive clinician review and interpretation (Fig. 1, Step 6c), constituting the final SRH output used for analysis.

### 2.4 Workflow assessment and image quality evaluation

Time from intraoperative tissue acquisition to availability of SRH images was recorded in three patients. Image quality was evaluated descriptively based on visibility of nerve fibers, delineation of fascicular architecture, identification of endoneurium, perineurium, epineurium and the presence of preparation-related artifacts such as shearing, squashing or freezing artifacts.

### 2.5 Ethics

Informed written consent was obtained from all patients. Ethical approval for tissue biobanking and analysis was obtained from the Ethics Committee of the University of Freiburg (approval numbers 23-1174-S1 and 23-1175-S1).

## 3 Results

**Fig. 2:**
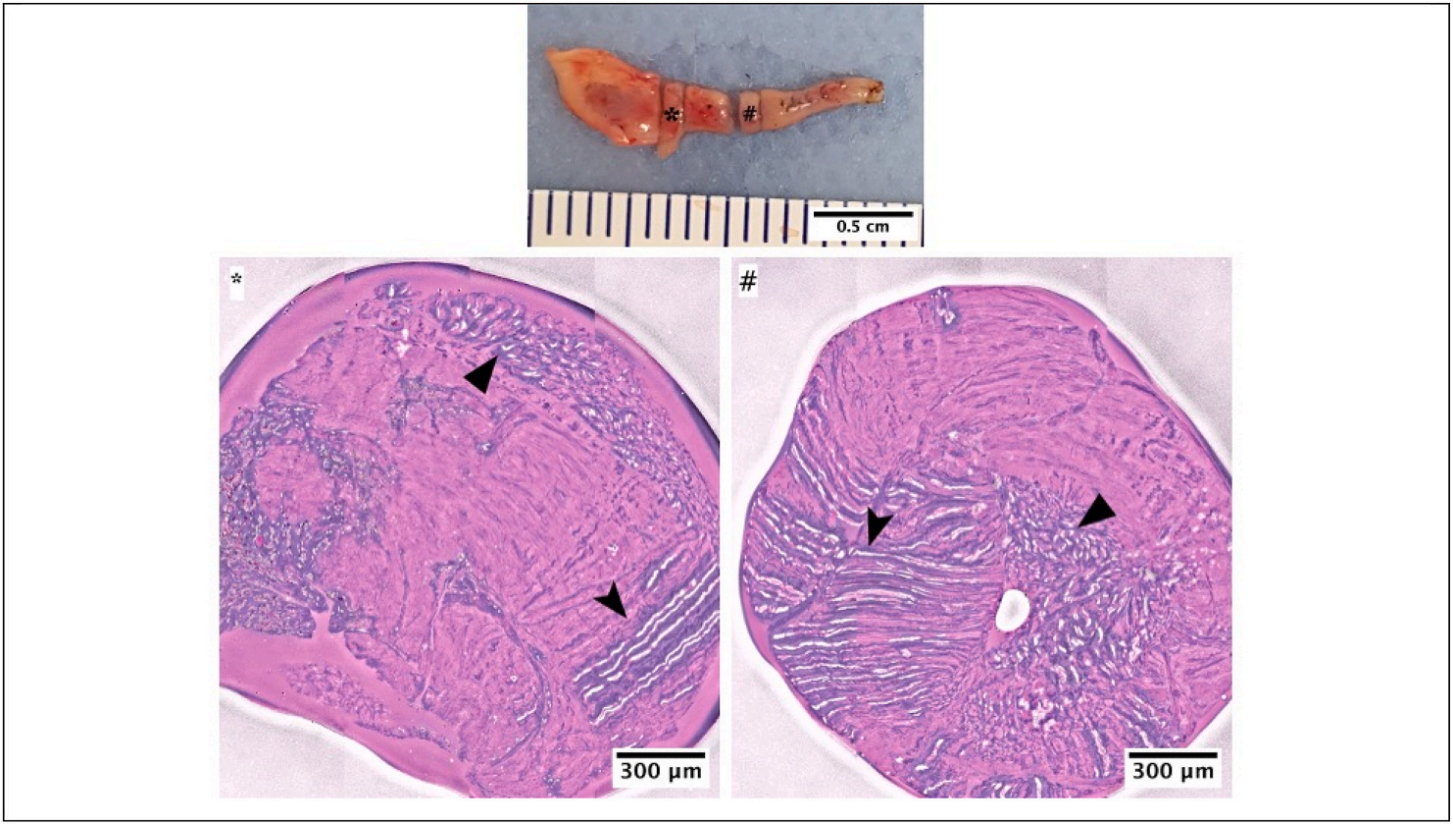
SRH of freehand-sectioned neuromatous tibial nerve fascicle (Sample 1). *Top:* Macroscopic view of the nerve fascicle. Two locations (*) and *(#)* indicate sites where freehand cross-sections (∼1 mm thickness) were obtained. Bottom left (*): SRH image of the squash preparation derived from the cross-section proximal to the neuroma. *Bottom right (#):* SRH image of the squash preparation derived from the cross-section distal to the neuroma. SRH allows clear identification of cross-sectioned nerve fibers (filled arrowhead) distinguishable from longitudinally oriented fibers (notched arrowhead). However, squash preparation of ∼1 mm-thick freehand sections results in marked architectural distortion and loss of true cross-sectional geometry, limiting reliable assessment of fascicular organization despite originating from the same fascicle.

Freehand sectioning of native nerve tissue without stabilization resulted in relatively thick specimens (∼1 mm or greater). When these samples were subsequently compressed to the required imaging thickness of 225 μm, substantial distortion of nerve microarchitecture occurred. SRH imaging of such preparations showed poor delineation of fascicular boundaries and connective tissue sheaths. The squash preparations consistently caused marked architectural distortion, including longitudinal displacement of nerve fibers and partial masking of fascicles by connective tissue layers, thereby precluding reliable and repeated cross-sectional assessment (Fig. 2).

**Fig. 3:**
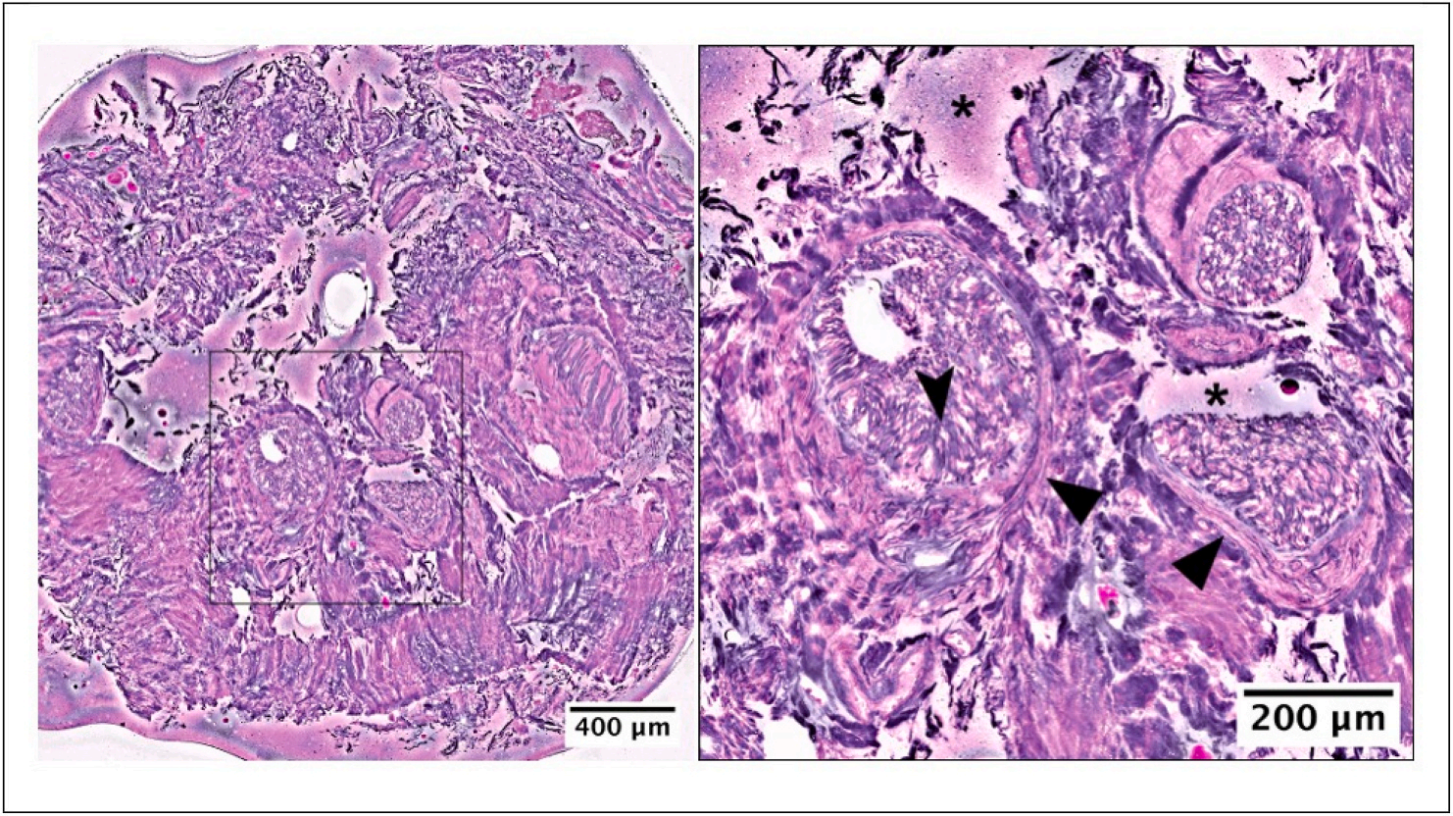
SRH of guided-sectioned native sural nerve (Sample 2). SRH image of a 500 μm-thick native sural nerve cross-section. Perineurium (filled arrowhead) and endoneurium of cross-sectioned nerve fascicles (notched arrowhead) can be distinguished. Shearing artifacts are visible (*).

Introduction of a metal cutting matrix (Fig. 1, Step 3) enabled guided thinner sectioning. In 10 cases, native nerve samples could be sectioned into approximately 500 μm–thick cross-sections, resulting in SRH images with clearly identifiable fascicles and fewer squash artifacts (Fig. 3). However, overall reproducibility remained limited. During sectioning within the cutting matrix, the soft and highly flexible specimen of native, unfixed nerve tissue tended to shift rather than to be cleanly transected. This resulted in imprecise cuts and made it difficult to reliably generate uniform cross-sections matching the predefined 500 μm spacing of the matrix.

**Fig. 4:**
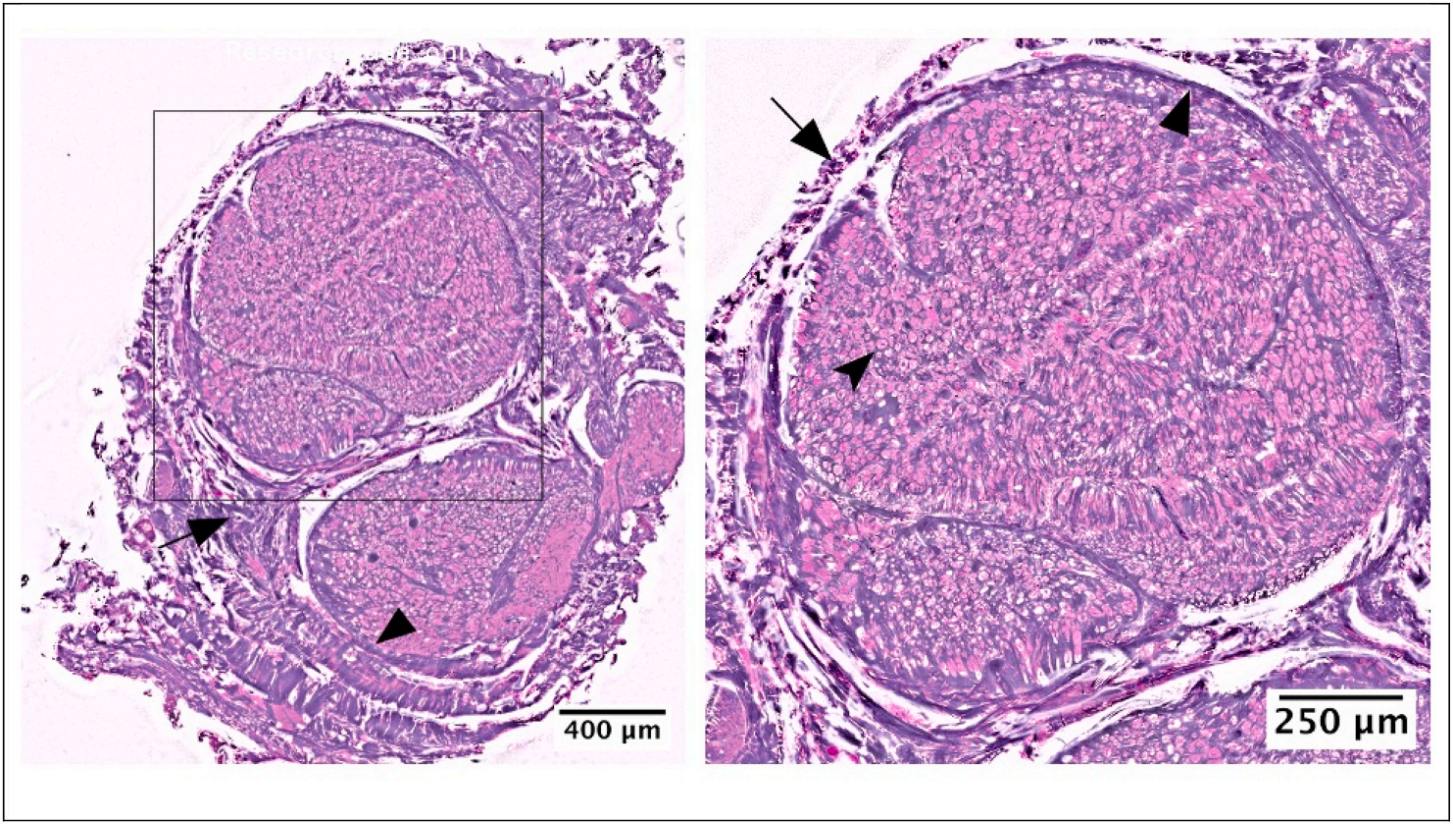
SRH of cutting matrix-assisted, guided-sectioned, formalin-fixed thoracic spinal nerve (Sample 12). SRH image of a 500 μm-thick formalin-fixed thoracic spinal nerve cross-section with preserved fascicular organization and minimal artifacts. Epineurium (arrow), perineurium (filled arrowhead), and endoneurium with nerve fibers (notched arrowhead) can be distinguished.

To enhance cutting precision tissue hardening by formalin fixation of the nerve specimen was explored. This allowed matrix-assisted reproducible generation of 500 μm thick cross-sections with well-preserved fascicular organization, clearly identifiable epineurial and perineurial structures, and the absence of relevant artifacts on SRH imaging (Fig. 4). Owing to prolonged processing time of several hours for chemical fixation–in this case 72 h–this approach was deemed unsuitable for intraoperative use.

**Fig. 5:**
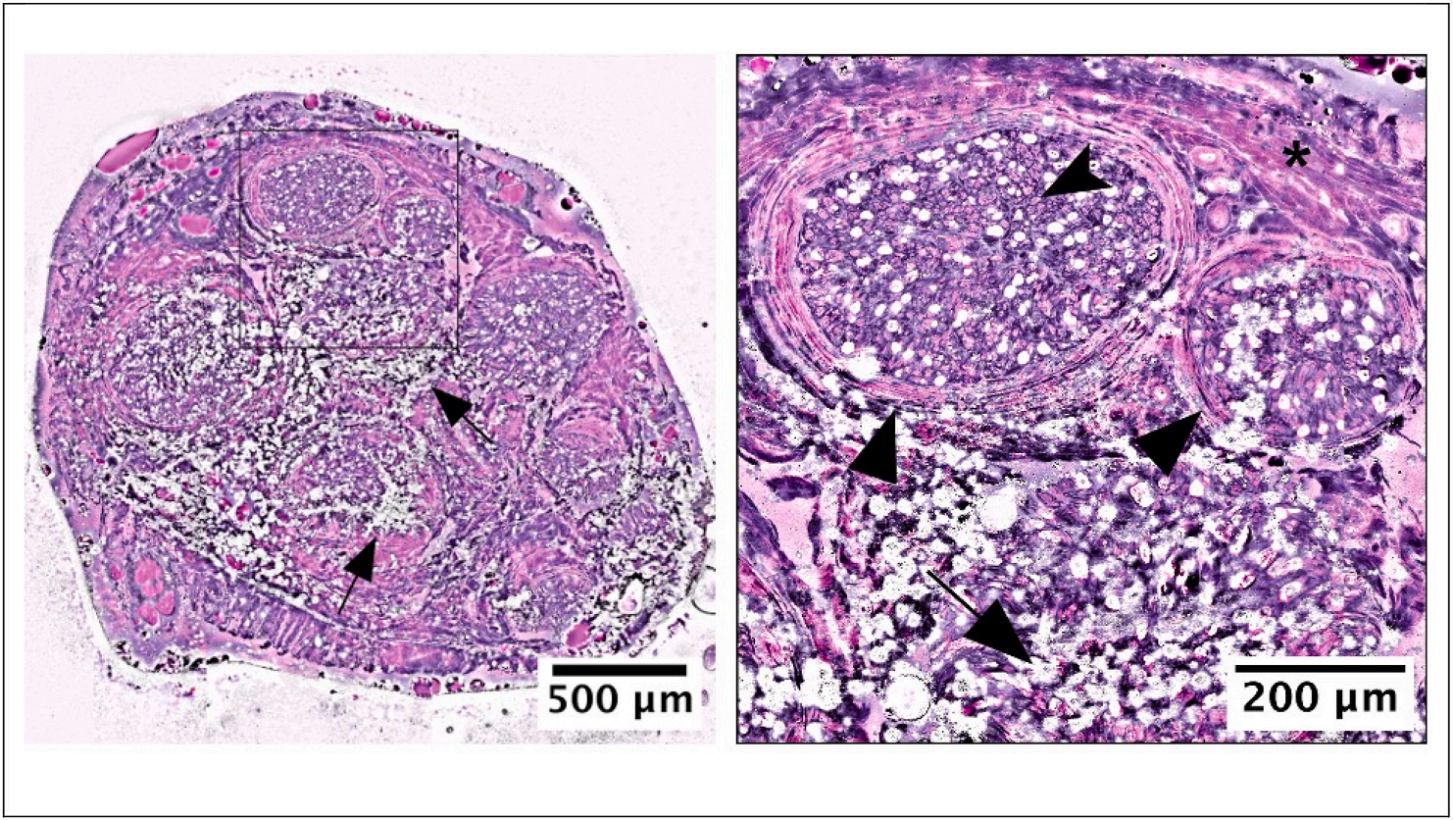
Frozen-agarose tissue preparation for SRH (FA-SRH) of sural nerve exposed to dry ice for 2 min (Sample 13). SRH image of a 500 μm-thick native sural nerve cross-section demonstrating preserved microarchitecture with freezing artifacts (arrow). *Right panel*: perineurium (filled arrowhead) and endoneurium with nerve fibers (notched arrowhead) are visible. Fascicles are surrounded by epineurium (*).

Therefore, short-term tissue hardening by controlled freezing was evaluated. Embedding native nerve tissue in agarose followed by dry ice exposure (n = 3, Fig. 1) provided sufficient mechanical stability to permit reproducible, controlled sawing-like sectioning without relevant deformation of the nerve tissue. This approach resulted in precise sectioning of uniform 500 μm-thick cross-sections suitable for SRH imaging. In one sample, a freezing duration of 2 min enabled sectioning but resulted in freezing artifacts on SRH imaging (Fig. 5).

**Fig. 6:**
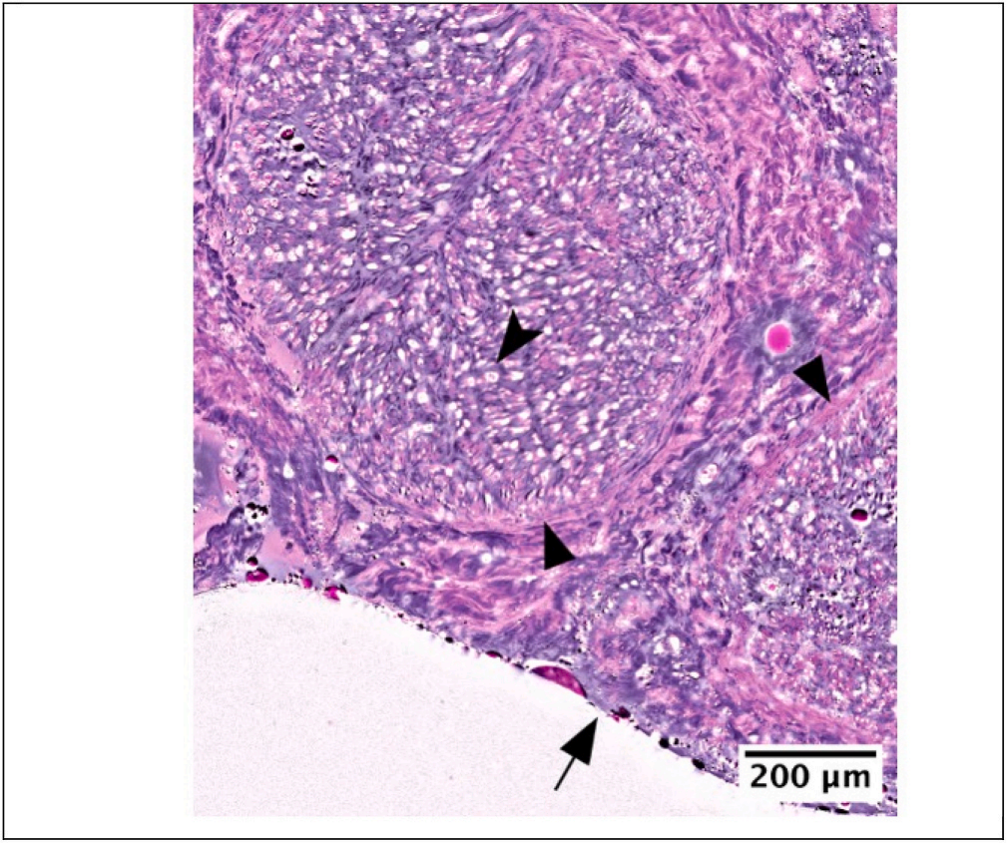
Frozen-agarose tissue preparation for SRH (FA-SRH) of sural nerve exposed to dry iced for 1 min (Sample 14). SRH image of a 500 μm-thick native sural nerve cross-section demonstrating preserved microarchitecture without freezing artifacts. Two fascicles surrounded by perineurium (filled arrowhead) within the epineurium (arrow) contain visible nerve fibers surrounded by endoneurium (notched arrowhead).

**Fig. 7:**
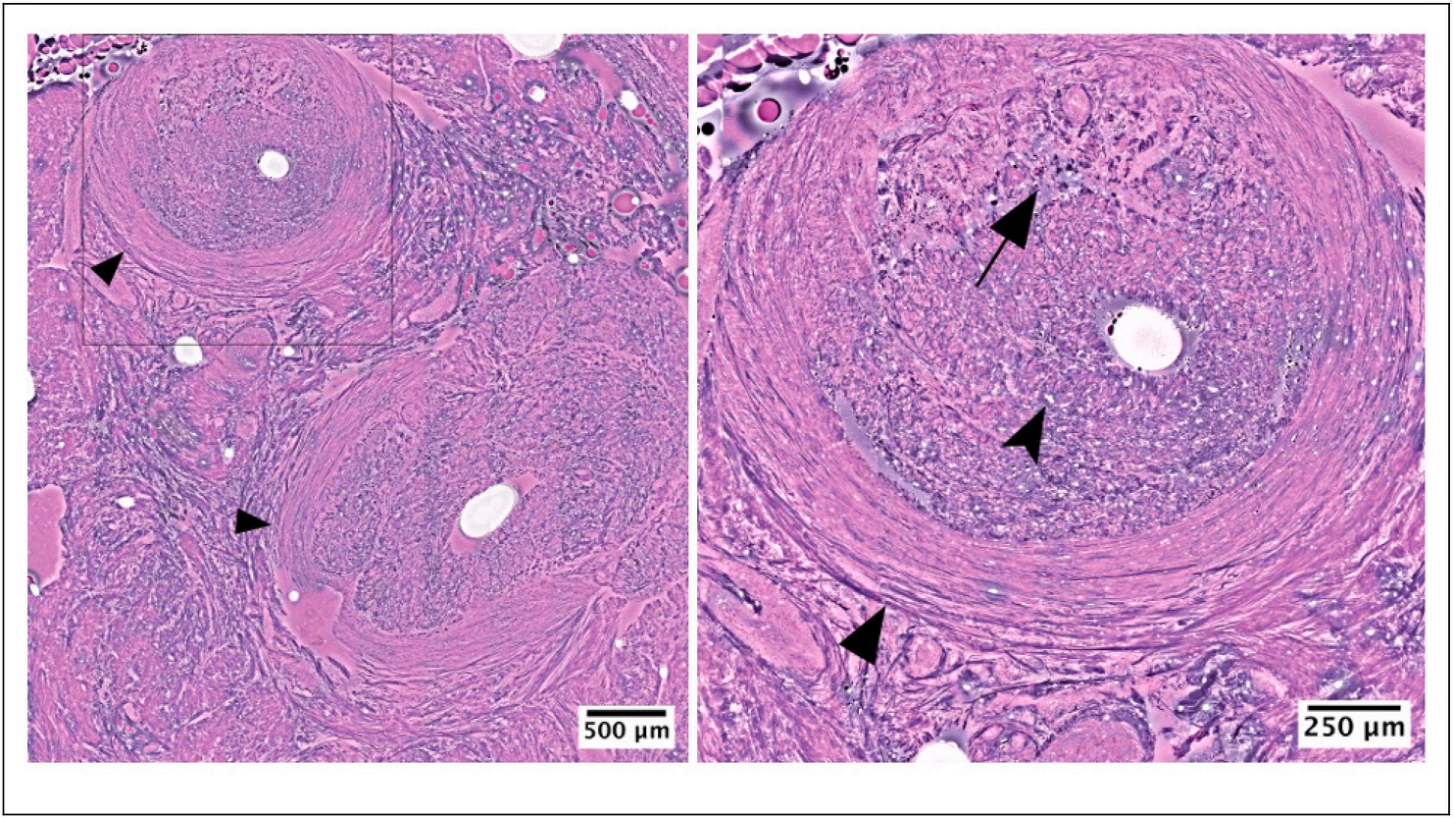
Frozen-agarose tissue preparation for SRH (FA-SRH) of guided-sectioned, peroneal nerve exposed to dry iced for 1 min (Sample 15). SRH image of a 500 μm-thick native neuromatous peroneal nerve stump cross-section demonstrating preserved microarchitecture without freezing artifacts. Two fascicles surrounded by perineurium (filled arrowhead) can be distinguished. The depicted fascicle on the right shows connective tissue (arrow) with only a few visible nerve fibers surrounded by endoneurium (notched arrowhead), which may indicate a neuromatous transformation of the nerve at this location.

The reduction of the exposure time to dry ice to 1 min eliminated these artifacts in two samples while preserving sufficient rigidity for cutting. SRH images demonstrated well-preserved fascicular architecture, identifiable connective tissue sheaths, and clearly visible nerve fibers without relevant squashing or freezing artifacts (Fig. 6, Fig. 7).

**Fig. 8:**
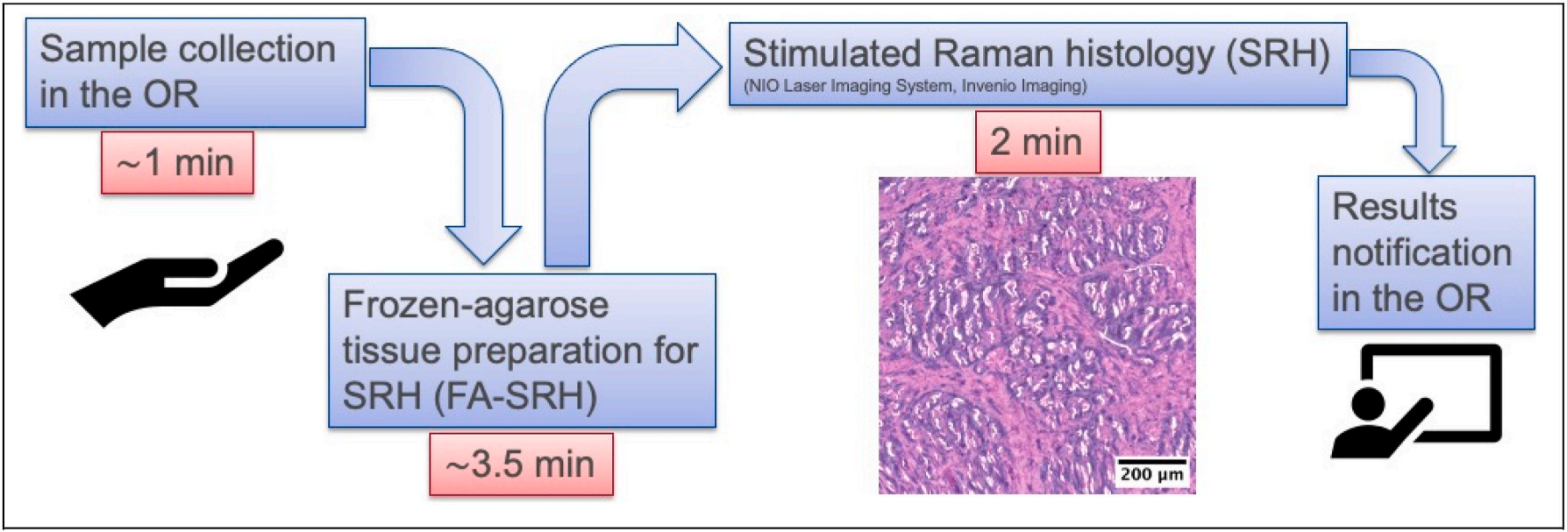
Frozen-agarose tissue preparation for SRH (FA-SRH) – intraoperative workflow. Optimized intraoperative SRH workflow for peripheral nerve assessment. Agarose embedding followed by 1 min of dry ice exposure enables reproducible generation of 500 μm cross-sections using a cutting matrix, resulting in a tissue preparation time of ∼3.5 min. Subsequent SRH imaging provides artifact-free visualization of fascicular architecture, connective tissue sheaths, and nerve fibers, allowing complete assessment of peripheral nerve tissue within ∼6–7 min depending on the selected field of view.

This optimized protocol finally resulted in a total tissue preparation time of approximately 3.5 min. Depending on the size of the field of view, this allowed the assessment of peripheral nerve tissue after approximately 6 to 7 min (Fig. 8).

## 4 Discussion

The FA-SRH approach demonstrates the feasibility of integrating SRH within a rapid intraoperative workflow allowing H&E-like objective visualization of peripheral nerve microarchitecture within approximately 6–7 min. FA-SRH allowed reliable visualization and judgement of fascicular organization and myelinated axons (Fig. 6, Fig. 7) and may enable intraoperative surgical decision-making during peripheral nerve repair surgery.

Accurate intraoperative assessment of peripheral nerve tissue remains a central challenge in the surgical management of nerve injuries, as the extent of neuromatous resection directly influences both the feasibility of tension-free reconstruction and the potential for functional recovery. In current clinical practice, this decision is largely based on subjective macroscopic criteria and the surgeon’s experience (Moore et al., 2015; Fones et al., 2024). As an objective criterion, a previous study demonstrated a marked heterogeneity in the expression of regeneration- and myelination-associated proteins in human neuromas, even within macroscopically similar nerve segments (Dömer et al., 2018). These findings indicate that visual and tactile assessment alone may not reliably reflect the underlying biological integrity or regenerative potential of a given nerve stump.

SRH provides rapid, label-free imaging with sufficient contrast to identify nerve fibers, fascicles, and connective tissue sheaths. Unlike in CNS tumors where SRH is widely used (Hollon et al., 2020; Straehle et al., 2022), tissue preparation emerged as the principal limiting factor for rapid intraoperative SRH of peripheral nerves. Inadequate and imprecise freehand sectioning resulted in substantial architectural distortion during squash preparations, leading to displacement of nerve fibers and masking of relevant structures, thereby limiting interpretability (Fig. 2).

By introducing a metal cutting matrix combined with agarose embedding followed by short-term tissue hardening over dry ice (FA-SRH), we were able to reproducibly generate thin (500 μm) nerve cross-sections suitable for SRH imaging within a clinically acceptable time frame. This fast FA-SRH protocol enabled consistent visualization of fascicular architecture and internal nerve fiber structure in macroscopically healthy and pathological peripheral nerve samples. Formalin fixation yielded excellent section quality (Fig. 4); its prolonged processing time, however, precluded intraoperative use.

From a workflow perspective, the total turnaround time of approximately 6–7 min from tissue acquisition to image availability represents a substantial advantage over conventional frozen section histology. While frozen section analysis remains the gold standard for definitive histopathological evaluation, reported turnaround times of up to 20 min and its limited suitability for repeated sampling restrict its routine application during sequential nerve stump trimming (Novis et al., 1997). In this context, SRH should be regarded as a complementary intraoperative tool rather than a replacement for conventional histopathology, addressing the specific need for rapid, objective microstructural feedback without disrupting operative flow.

The ability to objectively assess nerve stump integrity during surgery may help reduce both under- and over-resection, potentially improving conditions for successful nerve repair while minimizing defect length. Repeated SRH-based assessment during sequential trimming could enhance intraoperative confidence and reduce reliance on purely subjective criteria. Although the present study does not aim to establish diagnostic accuracy or to replace neuropathological evaluation, it provides a technical foundation for intraoperative SRH application in a clinically relevant setting.

The present study represents a feasibility study establishing a rapid and reproducible SRH workflow under realistic surgical conditions. It provides a basis for future evaluation across different peripheral nerve pathologies. Traumatic nerve injuries may be a promising application, as SRH could enable repeated assessment during sequential trimming to identify optimal coaptation levels. Further refinement and validation are required before clinical implementation.

Several limitations should be acknowledged. For the histological identification of nerve regeneration, a previous study suggests the ability to detect aberrantly sprouting axons in conventional histology (Malessy et al., 1999). While nerve fibers are visible in SRH images (Fig. 5, Fig. 6), this study did not include sufficient specimens with pronounced regenerative changes to systematically assess aberrant axonal sprouting. Future studies with well-characterized samples are needed.

Additional potential histological features for the assessment of nerve integrity are the organization of the endoneurium, the presence of endoneurial free space, as well as the definition and thickening of the perineurium (Murji et al., 2008). Furthermore, the overall myelin proportion has been shown to correlate with functional outcomes following nerve reconstruction (Malessy et al., 1999). In this context, identification of a structurally preserved nerve stump with an adequate myelin proportion may support selection of the resection level. SRH reliably visualized these features and may provide objective guidance during trimming.

Optimization of the tissue preparation protocol was performed in a limited number of samples, reflecting the exploratory nature of this feasibility study. While sufficient to establish a reproducible workflow, further validation in larger sample series will be required to confirm robustness across different nerve types and pathological conditions. Among the 15 lesions included in this study, only a subset represents conditions in which SRH imaging may have direct clinical relevance as an intraoperative tool in peripheral nerve surgery. In the context of schwannoma surgery, current textbook recommendations regarding resection margins are largely empirical and not well defined (Heinen et al., 2014). The technique presented here may offer the potential to objectively assess tumor-free fascicles at the resection margin and thereby contribute to a more quantitative definition of adequate margins. This aspect, however, remains to be addressed in future studies.

Direct comparison of SRH with conventional histological techniques was not performed and remains a subject for future studies. Prior studies demonstrated morphological concordance (Wilson et al., 2022). This study focused on intraoperative applicability, where SRH may fill a gap due to the time constraints of standard histology.

Image interpretation was qualitative and performed by investigators without formal neuropathological training, and interobserver variability was not assessed.

Importantly, while SRH does not directly interrogate molecular regeneration markers, its non-destructive nature represents a key translational advantage. Unlike conventional histological techniques, FA-SRH preserves tissue integrity and does not preclude subsequent downstream analyses. This enables post hoc molecular or immunohistochemical characterization of the same tissue sample. Future correlative studies linking SRH-derived microstructural features with molecular regeneration profiles may help bridge near real-time intraoperative imaging with underlying biological determinants of nerve regeneration.

Future work should focus on establishing comprehensive SRH image databases of healthy and pathological peripheral nerves, enabling systematic correlation with conventional histopathology and long-term functional outcomes following nerve reconstruction. Integration of machine learning–based image analysis may further enhance reproducibility, reduce observer-dependent variability, and facilitate standardization across centers (Hollon et al., 2020; Reinecke et al., 2022; Scheffler et al., 2025). Validation in larger, multicenter studies will be essential to define the clinical role of SRH-guided intraoperative assessment in peripheral nerve surgery.

## 5 Conclusions

FA-SRH, combining SRH with a rapid and reproducible tissue preparation protocol, is an easily reproducible adjunct for the intraoperative assessment of peripheral nerve tissue. By enabling reliable cross-sectional visualization of nerve microarchitecture within minutes, FA-SRH provides objective histology-like information that may support intraoperative decision-making during sequential nerve stump resection. FA-SRH is intended to complement established standards by addressing the need for rapid intraoperative feedback to improve conditions for successful nerve repair. Further prospective studies are required to define the clinical impact of FA-SRH-guided nerve repair on functional outcomes.

## Funding source

J.S. received funding from the Berta-Ottenstein Program for Clinician Scientists, 10.13039/501100021729Faculty of Medicine, University of Freiburg, Germany.

## Declaration of competing interest

The authors declare the following financial interests/personal relationships which may be considered as potential competing interests: Jakob Straehle reports financial support was provided by Berta-Ottenstein Program for Clinician Scientists, Faculty of Medicine, University of Freiburg, Germany. If there are other authors, they declare that they have no known competing financial interests or personal relationships that could have appeared to influence the work reported in this paper.

## References

[bib1] Aman M. (2022). An epidemiological and etiological analysis of 5026 peripheral nerve lesions from a European level I trauma center. J. Personalized Med..

[bib2] Dömer P. (2018). Analysis of regeneration- and myelination-associated proteins in human neuroma in continuity and discontinuity. Acta Neurochir..

[bib3] Dunn J.C. (2021). Supercharge end-to-side nerve transfer: systematic review. Hand.

[bib4] Fones L., Rivlin M., Tosti R. (2024). Cutting-edge approaches for nerve debridement prior to repair. J. Hand Surg. Glob. Online.

[bib5] Heinen C., Kretschmer T., Weis J., Kompression, Kretschmer T., Antoniadis G., Assmus H. (2014). Nervenchirurgie: Trauma, Tumor.

[bib6] Hollon T.C. (2020). Near real-time intraoperative brain tumor diagnosis using stimulated Raman histology and deep neural networks. Nat. Med..

[bib7] Kim D.H. (2004). Management and outcomes in 353 surgically treated sciatic nerve lesions. J. Neurosurg..

[bib8] Kim D.H. (2004). Management and outcomes in 318 operative common peroneal nerve lesions at the Louisiana State University Health Sciences Center. Neurosurgery.

[bib9] Kim J.Y. (2015). Surgical duration and risk of venous thromboembolism. JAMA Surg..

[bib10] Malessy M.J. (1999). Correlation between histopathological findings in C-5 and C-6 nerve stumps and motor recovery following nerve grafting for repair of brachial plexus injury. J. Neurosurg..

[bib11] Moore A.M., Wagner I.J., Fox I.K. (2015). Principles of nerve repair in complex wounds of the upper extremity. Semin. Plast. Surg..

[bib12] Murji A. (2008). The role of intraoperative frozen section histology in obstetrical brachial plexus reconstruction. J. Reconstr. Microsurg..

[bib13] Neidert N. (2022). Stimulated Raman histology in the neurosurgical workflow of a major European neurosurgical center - part A. Neurosurg. Rev..

[bib14] Novis D.A., Zarbo R.J. (1997). Interinstitutional comparison of frozen section turnaround time. A College of American Pathologists Q-Probes study of 32868 frozen sections in 700 hospitals. Arch. Pathol. Lab Med..

[bib15] Orringer D.A. (2017). Rapid intraoperative histology of unprocessed surgical specimens via fibre-laser-based stimulated Raman scattering microscopy. Nat. Biomed. Eng..

[bib16] Reinecke D. (2022). Novel rapid intraoperative qualitative tumor detection by a residual convolutional neural network using label-free stimulated Raman scattering microscopy. Acta Neuropathol. Commun..

[bib17] Scheffler P. (2025). Intraoperative classification of CNS lymphoma and glioblastoma by AI-based analysis of Stimulated Raman Histology (SRH). Brain Spine.

[bib18] Scholz C. (2023). Meralgia paraesthetica: relevanz, Diagnostik und Therapie. Dtsch Arztebl Int..

[bib19] Seddon H.J. (1942). A classification of nerve injuries. Br. Med. J..

[bib20] Straehle J. (2022). Neuropathological interpretation of stimulated Raman histology images of brain and spine tumors: part B. Neurosurg. Rev..

[bib21] Sunderland S. (1951). A classification of peripheral nerve injuries producing loss of function. Brain.

[bib22] Wilson T.J. (2022). Initial experience with label-free stimulated Raman scattering microscopy for intraoperative assessment of peripheral nerves. Clin. Neurol. Neurosurg..

